# Automatic Tuning of Gaussian Filter for Image Vignetting Correction

**DOI:** 10.3390/s26092648

**Published:** 2026-04-24

**Authors:** Artur Bal, Henryk Palus

**Affiliations:** 1 Department of Data Science and Engineering, Silesian University of Technology, Akademicka 16 Str., 44-100 Gliwice, Poland; 2 Biotechnology Center, Silesian University of Technology, Bolesława Krzywoustego 8 Str., 44-100 Gliwice, Poland

**Keywords:** image vignetting, vignetting correction, flat-field correction, Gaussian filter, automated filter tuning, lens–camera systems, polynomial modelling, low-level image processing

## Abstract

Image vignetting is a common optical artefact characterised by a gradual reduction in brightness towards the edges of an image. It degrades image quality and compromises radiometric accuracy, affecting a wide range of imaging applications. When vignetting cannot be avoided during acquisition, a computational correction is required. Existing methods often rely on Gaussian filtering of a flat-field image; however, selecting the appropriate filter parameters—particularly the standard deviation—remains challenging and is largely subjective. To address this issue, this paper presents the Gaussian filter with auto-tuned sigma (GFATS) method. This method leverages an optimisation framework to automatically tune filter parameters. This is achieved by aligning the filtered output with a specified polynomial model of the vignetting profile near the optical centre of the captured flat-field image. The tuned filter is then applied to the same image to derive the vignetting correction matrix. The proposed method was evaluated against established model-based correction methods across different lens–camera systems using objective quantitative measures. The results demonstrate that GFATS provides a more accurate vignetting estimation and improves the brightness uniformity of corrected images compared to existing approaches. It also effectively mitigates the overfitting issue inherent to standard low-pass filtering or smoothing methods. Notably, a single consistent parameter set was found to provide a reliable and stable performance across all tested lens–camera systems, demonstrating the method’s versatility and strong practical applicability.

## 1. Introduction

Image vignetting is a common optical artefact, characterised by a decrease in brightness from the centre to the periphery of an image as a function of distance. This phenomenon can be particularly detrimental to applications requiring accurate radiometric or quantitative analyses. Such applications include, e.g., astronomy [1,2], microscopy [3,4], remote sensing [5,6,7], multi- and hyperspectral imaging [4,8,9,10], and 3D mapping [11]. Vignetting also degrades computational imaging tasks such as high-dynamic-range (HDR) imaging [12], panoramic image creation [13,14] and mosaic reconstruction [15,16], as well as data-driven analyses performed using neural networks [17,18,19]. On the other hand, vignetting can be used for tasks like image authentication [20].

During image acquisition, vignetting can be reduced by selecting a smaller aperture, employing a longer focal length, or removing any superfluous filters. However, these approaches are not always feasible, and the complete elimination of vignetting is generally impossible. Therefore, a computationally based vignetting correction stage is, in practice, routinely performed as part of the image preprocessing pipeline. Historically, numerous different approaches to vignetting correction have been developed. These methods differ in terms of the type and volume of data required, their precision, and their specific operational requirements.

More recently, methods based on deep neural networks, notably generative adversarial networks (GANs), have advanced significantly. These techniques are primarily used to correct vignetting in acquired images when a dedicated reference image cannot be obtained, either due to a lack of access to the original imaging system or an unknown set of acquisition parameters [19,21,22,23]. Such approaches are also utilised when the system parameters are not constant during the image acquisition process [24,25]. However, the application of these methods typically results in a relatively imprecise vignetting correction, which limits their use in applications requiring the analysis of radiometric data retrieved from a scene—a common scenario in fields such as remote sensing.

A more traditional and widely adopted approach involves the determination of a vignetting model. This can be achieved through a priori knowledge of the optical system or by estimating vignetting parameters from a dedicated test image. For instance, various physics-based models have been developed to describe the processes occurring within the lens–camera system, accounting for different types of vignetting, such as natural vignetting [26,27] and optical vignetting [28]. While these models accurately describe the dependence of brightness on the distance from the image centre, they require detailed knowledge of the physical parameters of the lens and camera—information that is typically difficult for vision system users to obtain.

Most practical vignetting correction methods require acquiring a high-quality reference image, obtained either from uniform illumination falling onto the camera or from a scene with uniform luminance [26]. Such a reference image is used to approximate the vignetting function of the lens–camera system, which describes the influence of vignetting on the captured image. These methods are referred to in the literature as flat-field methods, and the corresponding reference image is termed a flat-field image. In the approximation process, parametric models are used (e.g., [29,30,31,32,33,34]), primarily polynomial ones, several examples of which are presented in Section 3. In some cases, the model requires assuming the coordinates of the pixel representing the image centre [35,36,37]. The coordinates of this point can be determined using additional methods [38,39], which complicates the process of estimating the vignetting function. Despite their advantages, polynomial-based vignetting estimation methods have well-recognised limitations, which become particularly apparent in imaging systems exhibiting irregular vignetting patterns. Consequently, relatively large vignetting correction errors often emerge, especially near the image edges, where the polynomial model may fail to capture complex local variations.

A common strategy for bypassing these limitations involves estimating vignetting from a flat-field image $I_{f}$ smoothed with a 2D low-pass Gaussian filter [8,40,41,42,43]. The results of this approach critically depend on the filter parameters, which, in discrete space, are the standard deviation σ and the kernel size *a* of the discrete filter G. The individual weights within the kernel matrix are determined as follows:

(1) $$G\left(h,l\right)=\frac{1}{2\pi \sigma^{2}}e^{-\frac{h^{2}+l^{2}}{2\sigma^{2}}},$$

where h,l∈Z, and $-\frac{a-1}{2}\leq h,l\leq \frac{a-1}{2}$, and *a* is an odd integer. Although parameter selection is crucial, the existing literature related to vignetting correction addresses it only briefly, generally emphasising the need for a trade-off between reproducing the vignetting profile and avoiding overfitting to noise [8].

Although methods for the automated selection of Gaussian filter parameters are extensively discussed in signal and digital image processing research, such solutions are not typically applied to flat-field-based vignetting correction. This discrepancy arises from the fact that the filtration objectives and signal properties considered in general processing tasks differ from those of flat-field images encountered in vignetting correction. As a result, methods from other domains often produce estimates that lack sufficient generalisation. Such filters fail to capture the actual vignetting profile accurately.

To the best of the authors’ knowledge, the only study addressing automatic Gaussian filter parameter selection specifically for the vignetting correction problem is the work by Cao et al. [44]. They proposed a criterion for selecting the optimal σ value based on the mean and standard deviation of the flat-field image $I_{f}$ before and after filtering. Although Cao et al. reported satisfactory results for near-infrared imagery, our experiments demonstrate that their criteria fail when applied to visible-light cameras. Crucially, our experiment revealed that, for the tested datasets, the proposed criterion is never satisfied. Consequently, it is impossible to determine the Gaussian filter parameters using the proposed method, which effectively precludes its practical application. Therefore, this method is not analysed further in this paper. The detailed experimental results justifying this decision are presented in Appendix A. These findings further imply that, for visible-light cameras, there is currently no dedicated method in the literature for automated Gaussian filter parameter selection for vignetting correction. This lack of a reliable solution prompted the development of the framework proposed here.

In this paper, a novel vignetting estimation method called the Gaussian filter with auto-tuned sigma (GFATS) is proposed. In this approach, the trade-off between accurately reproducing the vignetting profile and suppressing image noise is addressed through an optimisation framework. The parameter-tuning problem is solved by identifying filter parameters that produce Gaussian filtering results resembling those obtained using a specified polynomial model M for the central region of the $I_{f}$ image (i.e., the region near the image’s optical centre). Because M is fitted over a relatively small central image region—where vignetting varies the least—this model is usually a very accurate approximation of the image-vignetting profile in this region. Furthermore, due to the properties of polynomial approximation based on mean squared error minimisation, M serves as a maximum likelihood estimator for vignetting in the central image region. The employment of M as a reference for Gaussian filtering enables the transfer of certain statistical properties to the target vignetting estimator obtained via the GFATS method. Specifically, GFATS introduces a hybrid approach where a polynomial model acts as a stable reference to tune a Gaussian filter. This prevents the estimation errors at image edges typical of global polynomials while retaining their strong generalisation properties without a tendency towards overfitting.

The article is organised as follows. Section 2 describes the GFATS method in detail. Section 3 presents the experiment conducted on real data to evaluate the GFATS method in comparison to existing vignetting estimation methods. The resulting data and discussion are provided in Section 4, while Section 5 concludes the paper.

## 2. Presentation of the GFATS Method

### 2.1. General Assumptions and Preliminaries

The input data for the GFATS method is a flat-field image $I_{f}$ [45]. It is preferable that the $I_{f}$ image has undergone dark noise correction [46,47]. The camera response function (CRF) should be linear or linearised [48,49,50]. In the case of colour images, a vignetting correction typically involves applying a single vignetting estimate, derived from the luminance channel, to all R, G, and B channels [31,35,51,52]. However, for high-precision tasks where an exact radiometric correction is pivotal, the correction can be performed independently for each colour channel [53,54]. Such independent treatment is also necessary for embedded or low-cost imaging systems, where vignetting levels often vary significantly across spectral channels [11,55]. In such cases, treating all channels jointly based on luminance would introduce non-uniform colour casts.

### 2.2. Algorithm

The GFATS method consists of three major steps, namely:

- STEP 1: **Estimation of M** — estimating the assumed polynomial model M for the central region ${N_{C}}^{★}$ of the $I_{f}$ image;
- STEP 2: **Searching for** $\sigma^{★}$ — tuning the $\sigma^{★}$ parameter based on M;
- STEP 3: **Calculation of** $\hat{V}$ — computing the final vignetting estimate $\hat{V}$.

**Figure 1:**
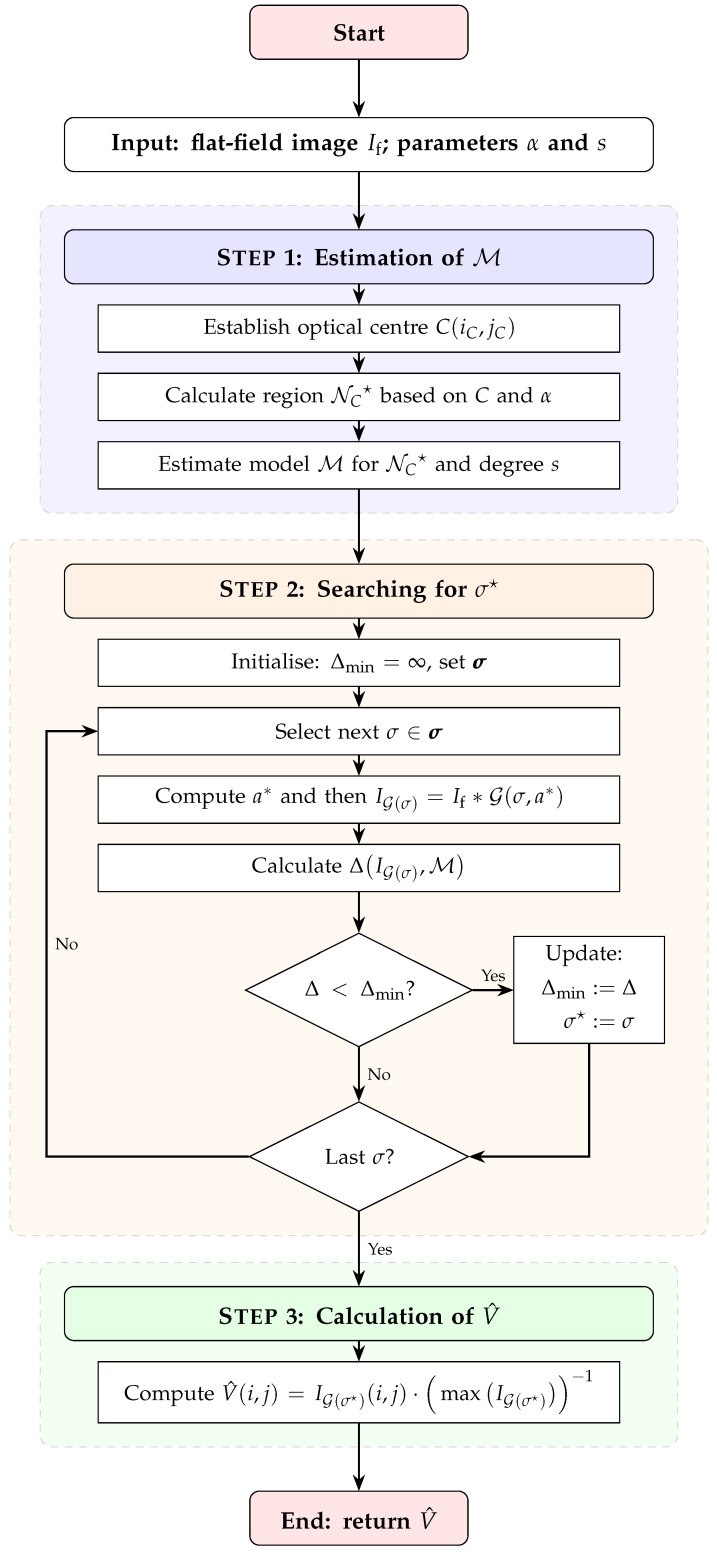
Flow chart of the GFATS algorithm with the exhaustive search procedure for determining the optimal $\sigma^{★}$ parameter in STEP 2.

The flowchart of the proposed algorithm is presented in Figure 1, illustrating the variant that employs an exhaustive search for the $\sigma^{★}$ parameter.

This approach leverages 2D polynomial approximation—a well-established technique in vignetting correction—to provide a good generalisation of the vignetting profile (model M) within the central region ${N_{C}}^{★}$ of the image $I_{f}$. In STEP 2, this model serves as a reference for determining the optimal $\sigma^{★}$ and the resulting Gaussian filter $G(\sigma^{★})$. The filter is tuned through an optimisation process to replicate the profile of the polynomial model M. Subsequently, in STEP 3, the $G(\sigma^{★})$ filter is applied to the entire image to compute the final vignetting estimate $\hat{V}$. By incorporating this reference, the Gaussian filter $G(\sigma^{★})$ inherits the strong generalisation properties of the polynomial model M. However, as the Gaussian filter operates locally, it maintains significantly greater flexibility than global polynomial models, which is particularly important in the peripheral regions of $I_{f}$. Consequently, the GFATS method effectively combines the primary advantages of both approaches, resulting in a reliable estimator that possesses a strong generalisation ability and resistance to overfitting.

STEP 1 **Estimation of** M

To estimate the polynomial model M of degree *s* (the first parameter of the GFATS method) for the central region ${N_{C}}^{★}$ of $I_{f}$, the coordinates $\left(i_{C},j_{C}\right)$ of the optical centre *C* of $I_{f}$ must be established. This is achieved by searching for the coordinates of the pixel $p\left(i,j\right)\in I_{f}$ where the second-degree 2D polynomial approximation of the image $I_{f}$ reaches its maximum.

The neighborhood $N_{C}\left(r\right)$ around centre *C* is defined by all pixels $p\left(i,j\right)\in I_{f}$ within a Euclidean distance D(C,p)≤r from *C*. Given the variable resolution M×N of the image $I_{f}$, it is practical to express the radius *r* as a function of the resolution through the parameter α:

(2) $$r^{*}=\min_{r\in 1,2,\ldots }(r:|N_{C}\left(r\right)|\geq \alpha MN),$$

where $\left|N_{C}\left(r\right)\right|$ denotes the cardinality of the set $N_{C}\left(r\right)$, representing the area of the region; $r^{*}$ is the radius of region ${N_{C}}^{★}$ used to calculate M; and α∈(0,1) is the desired fraction of the total pixels in $I_{f}$ to be included in the estimation. The coefficient α is the second and final parameter of the GFATS method. Finally, the model M is estimated for $p\left(i,j\right)\in {N_{C}}^{★}$ using a 2D polynomial approximation of degree *s*.

STEP 2 **Searching for**$\;\sigma^{★}$

As mentioned earlier, the discrete realisation of the Gaussian filter is defined by two parameters, σ and *a*. Because of the nature of the Gaussian filter, there is a practical limit of the $a^{*}$ value above which the filtration results for filters with the same σ and different values of *a* are virtually the same. The commonly used formula for calculating the $a^{*}$ value is as follows:

(3) $$a^{*}=2\;\cdot \;\left\lceil 3\sigma \right\rceil +1,$$

where ⌈·⌉ is the ceil function. By applying Equation (3), the task of tuning two separate parameters is reduced to a single-parameter optimisation of σ. From now on, let G(σ) denote a Gaussian filter with a standard deviation σ and the filter mask size $a^{*}$, which is calculated according to (3).

According to the GFATS method, the optimal $\sigma^{★}$ value is defined by the following condition:

(4) $$\sigma^{★}=\arg\min_{\sigma \in \sigma }\left(\Delta (I_{G(\sigma )},M)\right),$$

where σ is the set of all tested σ values, and $I_{G(\sigma )}=I_{f}*G\left(\sigma \right)$ denotes the result of the convolution of the image $I_{f}$ with the Gaussian filter G(σ). The term

(5) $$\Delta \left(I_{G(\sigma )},M\right)=\frac{1}{|{N_{C}}^{★}|}\sum_{p\left(i,j\right)\in {N_{C}}^{★}}{\left(I_{G(\sigma )}\left(i,j\right)-M\left(i,j\right)\right)}^{2}$$

represents the mean squared error (MSE) between the model M and the filtered image $I_{G(\sigma )}$ for a given σ.

STEP 3 **Calculation of**$\;\hat{V}$

The vignetting estimate $\hat{V}$ is calculated as follows:

(6) $$\hat{V}\left(i,j\right)=\frac{I_{G(\sigma^{★})}\left(i,j\right)}{\max\left(I_{G(\sigma^{★})}\right)},$$

where $I_{G(\sigma^{★})}=I_{f}*G\left(\sigma^{★}\right)$. The vignetting estimate matrix $\hat{V}$ is the result of the GFATS method.

The matrix $\hat{V}$ can be directly used for the vignetting correction of acquired images *I* using the following formula:

(7) $$\hat{I}(i,j)=\frac{I(i,j)-I_{\mathrm{DN}}(i,j)}{\hat{V}(i,j)},$$

where $\hat{I}$ is the image with corrected vignetting and $I_{\mathrm{DN}}$ is the dark image acquired under the same exposure conditions (i.e., exposure time, sensor’s sensitivity) as used during the acquisition of the *I* image. However, when the image $I_{\mathrm{DN}}$ has not been acquired, the simpler version of (7), that is,

(8) $$\hat{I}(i,j)=\frac{I(i,j)}{\hat{V}(i,j)}$$

can be used.

### 2.3. Implementation Details

To ensure the numerical stability of determining model M, and given the possibility of relatively large values occurring in the *x* and *y* coordinates, it is advisable to perform the approximation using normalised coordinates rather than the original values. Considering the nature of the input data, an appropriate solution is to apply min-max normalisation to the range of [−1,1] according to the formulae:

(9) $$\begin{array}{cc} x_{\mathrm{norm}} & =\frac{2x-(x_{\max}+x_{\min})}{x_{\max}-x_{\min}},\\ y_{\mathrm{norm}} & =\frac{2y-(y_{\max}+y_{\min})}{y_{\max}-y_{\min}}, \end{array}$$

where:

- x,y are the original pixel coordinates of the input images;
- $x_{\mathrm{norm}},y_{\mathrm{norm}}$ are the resulting normalised coordinate values;
- $x_{\min},y_{\min}$ and $x_{\max},y_{\max}$ are, respectively, the minimum and maximum values of the original set of coordinates.

Applying Gaussian filtering in the spatial domain presents difficulties with boundary effects, especially given the high-intensity gradients at image edges. Due to the large kernel sizes required, frequency-domain implementation via a fast Fourier transform (FFT) is preferred to ensure numerical stability and prevent boundary artefacts.

The optimisation of the parameter σ (STEP 2) can be approached in several ways. While an exhaustive search over a predefined set of values σ is the simplest to implement, it is computationally expensive due to the repeated convolutions required. A more efficient alternative is the use of derivative-free optimisation (DFO) techniques, such as the Nelder–Mead method, which significantly reduces the number of required iterations, thereby decreasing the overall computational time of the algorithm.

## 3. Experimental Comparison of Vignetting Estimation Methods

### 3.1. General Assumptions

The aim of this experiment was to compare, using real-world data, the GFATS vignetting estimation method with three model-based methods known from the literature:

- The deformable radial polynomial (DRP) model [34];
- The polynomial 2D approximation (P2D) model [29,31];
- The smooth non-iterative local polynomial (SNILP) model [32].

In terms of the estimation quality, these methods differ primarily in their ability to fit the analysed $I_{f}$ images. The DRP model is the least flexible, as it enforces axial symmetry on the resulting vignetting estimate $\hat{V}$, allowing for only limited deformation. The P2D model offers greater flexibility than the DRP. Finally, the SNILP model provides an even better fit to the $I_{f}$ image than its P2D counterpart.

As mentioned in Section 1, the comparison does not include the method proposed by Cao et al. [44]. This omission is a result of experimental evidence (see Appendix A) confirming the unsuitability of this method for determining the $\sigma^{★}$ parameter for cameras operating in the visible light spectrum, which were used in this experiment.

As the primary objective of the experiment was to compare the intrinsic properties of the vignetting estimation methods—rather than to achieve exact radiometric correction for specific lens–camera systems—the evaluation was conducted solely using luminance data. This approach remained consistent with the methodology outlined in Section 2.1. A detailed description of the experimental conditions is provided in the following subsections.

### 3.2. Experimental Conditions

#### 3.2.1. The Lens–Camera Systems

The images necessary to conduct the experiment were acquired with three different lens–camera systems, representing three currently widely used types of imaging systems, i.e., webcams and embedded-system cameras (WCam), systems used in machine vision and industrial applications (ICam), and digital photographic cameras (DCam). The major parameters of the used systems are presented in Table 1.

#### 3.2.2. Laboratory Set-Up

**Figure 2:**
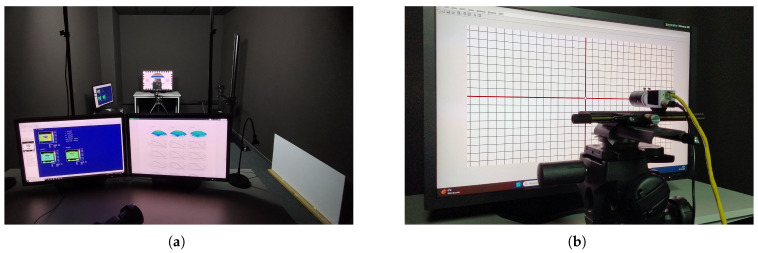
General view of the darkroom where the experiment was carried out (**a**) and the laboratory setups used for camera positioning (**b**). The lighting in the laboratory was switched on only for the purpose of taking the photographs shown.

Image acquisition was performed in a darkroom (Figure 2a), ensuring that random light sources did not influence the experimental results. A flat-field surface (flat scene) was created using a uniformly backlit, milky poly(methyl methacrylate) (PMMA, “plexiglass”) panel, measuring 50 cm×100 cm and 3 mm in thickness. A NEC SpectraView Reference 301 graphic monitor, displaying a white screen with a brightness of $300\;\mathrm{cd}/m^{2}$, served as the light source. During acquisition, the plexiglass panel was carefully positioned parallel to the monitor screen, with a constant distance of approximately 12 cm maintained throughout the acquisition process.

To ensure that the lens–camera system was parallel to the monitor, each system was centred relative to the screen. Its position and orientation were precisely adjusted until the geometric distortions in the captured calibration image (see Figure 2b) were perfectly symmetrical. The calibration pattern was displayed on the monitor used for panel illumination, while the acquired images were monitored in real time on a second display. During this alignment process, the plexiglass panel was removed to ensure unobstructed positioning.

#### 3.2.3. Image Acquisition and Processing

The vignetting estimation methods were evaluated using flat-field images captured by each camera. For every device, a set of images $I_{f_{k}}$ was acquired under identical conditions. The exposure parameters for the Logitech (WCam) and Basler (ICam) cameras were selected automatically. In contrast, the parameters for the Canon camera (DCam) were adjusted manually to ensure the absence of overexposed pixels. Images from the WCam and ICam were acquired directly to a computer using dedicated software (see Section 3.2.6). In the case of the DCam, the images were recorded directly onto an SD card using the camera’s internal storage.

To mitigate the influence of random noise, the images were first averaged. Specifically, the number of averaged frames was set to K=10 for the DCam, K=25 for the ICam, and K=100 for the WCam. The averaging process is defined as:

(10) $$\bar{I}=\frac{1}{K}\sum_{k=1}^{K}I_{f_{k}}.$$

Following this, dark noise correction was applied to the images from the WCam and DCam by subtracting their respective averaged dark frames.

Additionally, for both of these cameras, outlier removal was applied to the averaged images $\bar{I}$ to mitigate artefacts caused by sensor defects. For this purpose, a locally applied Tukey’s criterion was employed, specifically tuned to eliminate “far out” values. The identified pixels were then replaced with the median value of their immediate neighbourhood.

By design, the ICam averaged image $\bar{I}$ is monochrome, and thus directly represents the luminance of the image, allowing for the direct assignment $I_{f}:=\bar{I}$. For WCam and DCam, the final greyscale image $I_{f}$, which also corresponds to the luminance channel, is computed as follows:

(11) $$I_{f}=0.299{\bar{I}}_{R}+0.587{\bar{I}}_{G}+0.114{\bar{I}}_{B},$$

where ${\bar{I}}_{R}$, ${\bar{I}}_{G}$, and ${\bar{I}}_{B}$ denote the red, green, and blue channels of $\bar{I}$, respectively.

Table 2 summarises selected parameters characterising the $I_{f}$ images obtained for the investigated object–camera systems. From the perspective of vignetting correction, the most relevant parameters are those describing:

- The overall vignetting level υ, which is roughly estimated using the following formula:
  (12) $$\upsilon =\frac{\max\left(I_{f}\right)-\min\left(I_{f}\right)}{\max\left(I_{f}\right)};$$
- The non-radiality level of the vignetting η, which is calculated according to η [34].

For the tested images, υ ranged from approximately 0.1 (WCam) to over 0.33 (DCam). Similarly, the η values varied from η≈1 (DCam), indicating nearly ideal radial vignetting, to η≈1.3 (ICam), which exhibited strong non-radial vignetting along the horizontal axis.

The obtained $I_{f}$ images also differed significantly in their noise levels. Regarding the two disparity metrics used for the $I_{f}$ noise evaluation—the standard deviation (STD) and interquartile range (IQR)—a more than 4-fold difference was observed between the WCam and DCam, while a more than 3.5-fold difference was noted between the ICam and DCam. This diversity in image properties enhances the reliability of the comparison, as it facilitates the validation of methods across a broad spectrum of image characteristics.

**Table 2:** The main parameters of the $I_{V}$ images used in the experiment.

Parameter		Lens–Camera Set		
		WC_AM_	IC_AM_	DC_AM_
Image resolution		1920×1080	1280×960	5184×3456
*C*	$i_{C}$	877	617	2580
	$j_{C}$	539	482	1903
υ		0.1106	0.1617	0.3041
η		1.1944	1.2809	1.0193
$\mathrm{STD}\left(I_{f}\right)$		3.3329	3.8252	13.5503
$\mathrm{IQR}\left(I_{f}\right)$		5.0110	5.5600	20.6000

#### 3.2.4. Performance Evaluation of Vignetting Estimation Methods

To evaluate the quality of the vignetting estimation $\hat{V}$, two dispersion measures were used: the standard deviation (STD) and the interquartile range (IQR). Both measures were computed on the corrected images ${\hat{I}}_{f}$, defined by:

(13) $${\hat{I}}_{f}=\frac{I_{f}}{\hat{V}}.$$

Lower values of the STD and IQR indicate a more effective vignetting correction.

Whilst the STD reflects the overall dispersion of pixel values, the IQR is robust to outliers, as it remains unaffected by values beyond the $Q_{1}$ and $Q_{3}$ thresholds. In vignetting correction—where significant errors frequently occur in peripheral regions—the IQR provides a more reliable evaluation by focusing on the central region of the corrected image ${\hat{I}}_{f}$, which is typically of the greatest practical importance.

Additionally, the coefficient of determination $R^{2}\in [0,1]$ was computed. Values of R^2^ closer to 1 indicate that the estimated vignetting $\hat{V}$ better accounts for the variability present in the original image $I_{f}$.

#### 3.2.5. Parameter Selection for the Evaluated Methods

A common parameter across all the compared methods was the degree *s* of the polynomials used for estimation. For all the evaluated methods, s∈{2,3,…,12} was adopted. In addition to *s*, the GFATS method requires an α parameter; for comparison purposes, its performance was evaluated across six different values: 0.01, 0.025, 0.05, 0.1, 0.25, and 0.5.

Regarding the DRP method [34], a version was employed in which the estimation of the optical centre coordinates *C* was integrated into the vignetting estimation process; consequently, no additional parameters were required.

In the experiment, an implementation of the GFATS method was used in which an exhaustive search was employed instead of a derivative-free optimisation method (as suggested in Section 2.3). This approach was chosen due to the investigative nature of this comparison and the need to analyse the behaviour of the $\Delta (I_{G(\sigma )},M)$ function across the entire range of tested σ values. The set σ of analysed values was defined as:

(14) σ∈σ={0.5,1,1.5,…,100}.

#### 3.2.6. Software Environment

All calculations related to image processing and the performance comparison were conducted using dedicated scripts developed in MATLAB R2024a (Update 3), utilising the image processing, image acquisition, and optimisation toolboxes. Gaussian filtering was performed using the imgaussfilt function.

The MATLAB environment was also employed to develop the software for image acquisition using WCam and for displaying the calibration patterns required for camera alignment. For ICam, image acquisition was performed using the Basler Pylon 8.02 software.

## 4. Results and Discussion

### 4.1. Results

The quantitative evaluation of the compared methods, based on the STD, IQR, and R^2^ measures, is summarised in Table 3, Table 4, and Table 5 for WCam, ICam, and DCam, respectively. Additionally, Table 6, Table 7 and Table 8 provide the $\sigma^{★}$ values determined using the GFATS method for the tested lens–camera systems across the examined range of the α parameter.

**Figure 3:**
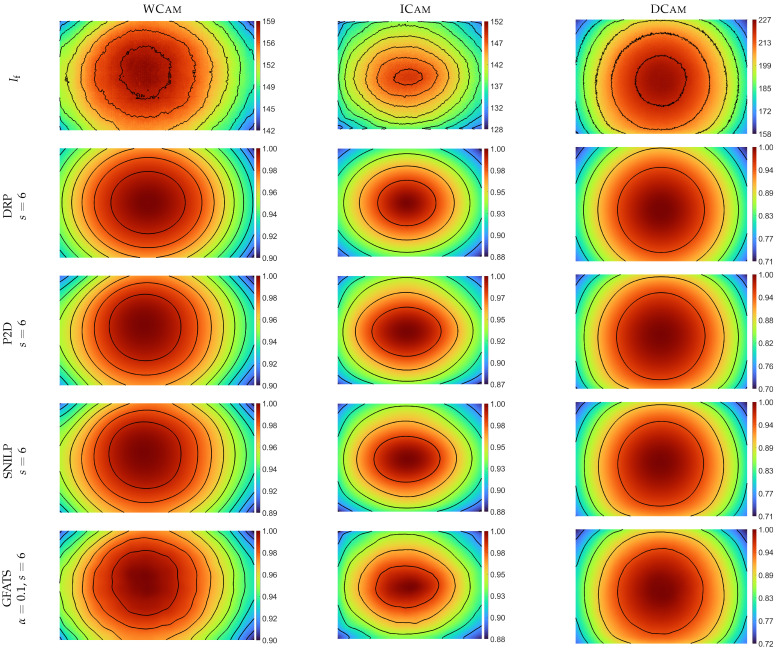
Vignetting estimation results $\hat{V}$ (rows 2–5) for s=6 (all tested methods) and α=0.1 (GFATS) compared against the original $I_{f}$ images (top row), with each column representing results for an individual lens–camera system.

To visualise and compare the performance of the tested methods, Figure 3 presents the contour plots of the $I_{f}$ and $\hat{V}$ images for a representative set of parameters: s=6 for the model-based methods, and s=6 and α=0.1 for the GFATS method. This type of plot allows for an easy assessment of how closely the analysed estimation methods adapt to the geometry of the input $I_{f}$ image.

**Figure 4:**
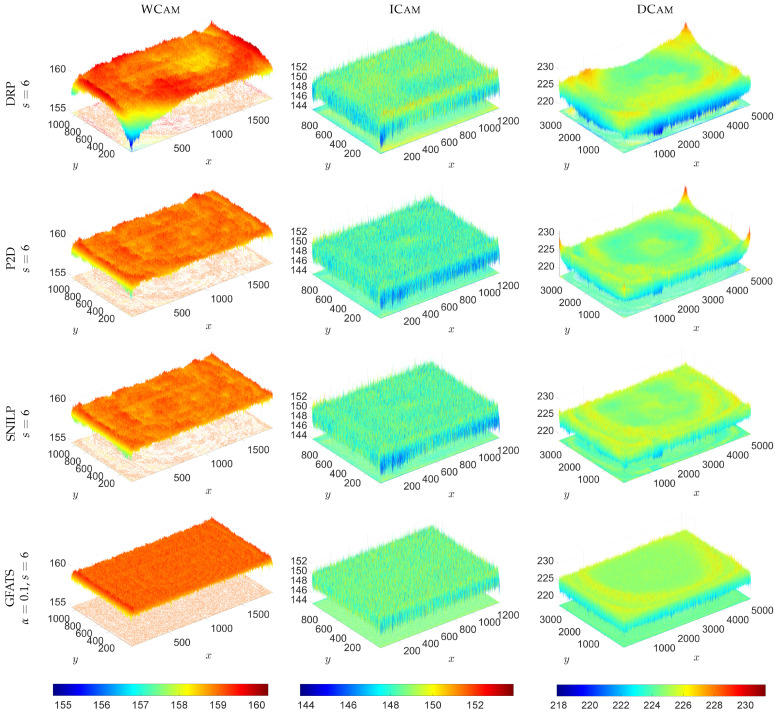
Comparison of vignetting correction results ${\hat{I}}_{f}$ for the tested vignetting estimation methods at s=6 (DRP, P2D, SNILP, GFATS) and α=0.1 (GFATS) across the tested lens–camera systems. For ease of comparison, the results for each system are arranged in columns using a consistent *Z*-axis range and colour scale (provided for each system in the bottom row).

Furthermore, Figure 4 displays the 3D plots of the obtained ${\hat{I}}_{f}$ images, providing a spatial perspective on the correction quality. For a fair and consistent comparison, the same color scale and Z-axis range were applied to all the results for each individual lens–camera system, presented here in columns. This visualisation approach is particularly useful for identifying any residual non-uniformities or local artefacts that might remain after the correction process. Using this approach, a perfect correction is represented by a perfectly flat surface with uniform coloration across the entire sensor area. Any remaining curvature or significant value fluctuations, visible as color variations, directly highlight the estimation errors of the respective methods. Comparing the magnitude of these fluctuations and the uniformity of colouration enables a direct evaluation of the estimation effectiveness. This approach facilitates a quick and intuitive performance assessment of each imaging system.

### 4.2. Discussion

#### 4.2.1. Estimation Performance and Flexibility

The obtained results indicate that, for each of the tested systems, the GFATS method provides more accurate vignetting estimation results than the compared model-based methods for the same polynomial order *s*. This high estimation quality is maintained largely independently of the chosen α parameter for a fixed value of *s*. Only for the ICam system, with α≥0.15 and s≥10, does the SNILP method provide slightly better results than the GFATS method.

The evaluation of the estimation results using the STD, IQR, and R^2^ metrics, combined with a comparison of the contours of $\hat{V}$ and $I_{f}$ (Figure 3), demonstrates that the GFATS method generally offers a greater flexibility in fitting $\hat{V}$ to the $I_{f}$ image compared to model-based approaches. Based on these results, the tested vignetting estimation methods can be ranked by their flexibility, ranging from the least flexible DRP, through P2D and SNILP, to the most flexible GFATS. This flexibility progression is particularly evident when comparing the shapes of the isolines (Figure 3). Progressing from DRP through P2D and SNILP to the GFATS method, the isolines align increasingly closely with those plotted for the corresponding $I_{f}$ images.

These findings suggest that GFATS serves as a bridge between model-based methods—whose fit is globally constrained by the chosen model and assumptions—and vignetting estimation via Gaussian filtering or other smoothing-based approaches. While the latter can produce results nearly identical to the input image if an inappropriate parameter is selected, they often fail to provide a sufficient generalising function, leading to the preservation of unwanted local noise or artefacts. In the GFATS method, the search for an optimal $\sigma^{★}$ to replicate the assumed model M as closely as possible ensures that the estimator maintains its required generalisation properties. Simultaneously, because the Gaussian filter—initialised with the $\sigma^{★}$ determined through this tuning process—is applied locally, a relatively high degree of estimation flexibility is preserved.

This observation indicates that the GFATS method is particularly well-suited for vignetting correction in systems where the vignetting profile is irregular or characterised by abrupt changes.

#### 4.2.2. Robustness to Overfitting

**Figure 5:**
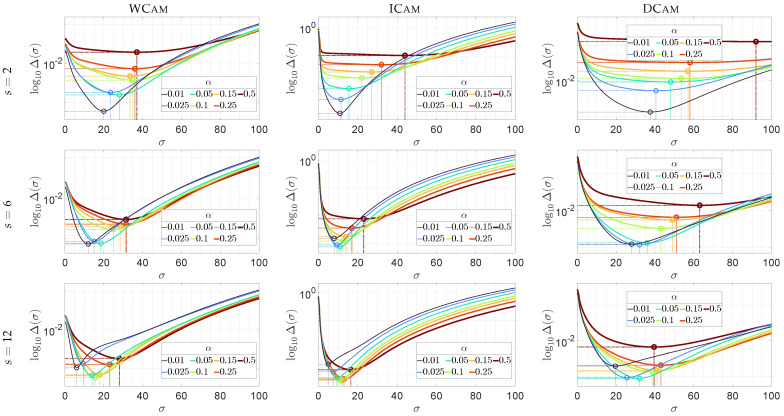
Profiles of the $\Delta (I_{G(\sigma )},M)$ function (Equation (5)) versus σ for polynomial model M of degree s=2,6, and 12 (top to bottom rows, respectively) across the evaluated lens–camera systems (columns).

The application of model M influences more than just the generalising properties of the GFATS method; it also effectively mitigates the risk of overfitting. This property is best illustrated by the plots of the $\Delta (I_{G(\sigma )},M)$ values (Equation (5)) as a function of σ (Figure 5) for various sets of parameters α and *s*.

As can be observed, all the curves possessed a well-defined, single global minimum. The value of σ at which this extremum occurs for each curve determines the optimal value $\sigma^{★}$ for the given set of α and *s*. This mechanism arises from the relationship between the reference model M and the results of using a Gaussian filter on region ${N_{C}}^{★}$ with parameter σ. When $\sigma <\sigma^{★}$, the filtering result overfits to local noise fluctuations in image $I_{f}$ compared to the more stable model M, leading to a higher $\Delta (I_{G(\sigma )},M)$ value than in the case of using $\sigma^{★}$. Conversely, when $\sigma >\sigma^{★}$, the filter excessively smooths the image, representing the vignetting profile less accurately than the reference model M, which again results in an increased value of $\Delta (I_{G(\sigma )},M)$. Consequently, the minimum of the objective function effectively identifies the balance where the filter best replicates the reliable characteristics of vignetting in the ${N_{C}}^{★}$ region of $I_{f}$ as provided by M.

It is important to note that, in every case, this extremum consistently occurs for σ values significantly larger than the minimum value in the considered set σ (in the experimental case, min(σ)=0.5). Such behaviour differs markedly from systems prone to overfitting, where values at or near the lower bound of the considered range would typically be selected as optimal. The absence of this effect in the GFATS method confirms that the proposed approach does not exhibit such a tendency. The absence of overfitting is further evidenced by the increase in $\sigma^{★}$ with α. This indicates that the GFATS method correctly scales its smoothing—and thus, its generalisation ability—to the size of the central region ${N_{C}}^{★}$ of the image $I_{f}$ utilised for the calculation of model M.

#### 4.2.3. Parameter Selection

The obtained STD and IQR values indicate that the performance of the GFATS method depends on the selection of the α and *s* parameters. However, this dependency varies across individual lens–camera systems. For each system, this variability can be evaluated using the maximum relative deviation, defined as:

(15) $$D\left(\mathrm{MES}\right)=\frac{d(\mathrm{MES})}{\min(\mathrm{MES})}\;\cdot \;100\%,$$

where **MES** denotes the set of all analysed values for a given measure MES, and

(16) d(MES)=max(MES)−min(MES)

is the maximum absolute deviation of measure MES. Table 9 and Table 10 present the spread values for the STD and IQR measures, respectively.

For instance, considering the spread of the STD measure across the entire analysed range of α and *s*, the D(STD) values for the GFATS method range from approximately 3.5% for the ICam system and about 10% for DCam, to over 32% for WCam. It should be noted, however, that when using the P2D and SNILP methods, the D(STD) variability is significantly higher—ranging from approximately three to over four times higher than in the case of the GFATS method. While D(STD) is a relative measure, the GFATS method also demonstrates a superior stability in terms of the absolute difference d(STD). In the worst-case scenario (the DCam system), this absolute difference for GFATS is less than 0.07, whereas for the P2D and SNILP methods, these differences are over three times larger.

In contrast to the previously discussed methods, the DRP method exhibits a much lower relative variation D(STD) than GFATS. This effect results from the DRP model’s limited fitting capabilities, which stem from its reliance on a radial vignetting function. While this constraint is somewhat relaxed in the DRP method by allowing for certain deformations, its influence remains significant. Such constraints prevent the model from capturing complex changes in the vignetting profile; consequently, its low variability is indicative of an inability to adapt to the data rather than evidence of estimation stability. Nevertheless, the DRP model remains capable of capturing the primary trend, providing a simplified representation of the overall vignetting structure.

An analysis similar to that carried out for the STD measure can be performed for the IQR measure (Table 10). In both cases, the results lead to the same conclusion: for the GFATS method—unlike the P2D and SNILP methods—the influence of the method’s parameters on the quality of the estimation results is relatively limited.

This finding relates to a key aspect of the practical application of estimation methods: the selection of their parameters. From this perspective, the results obtained highlight a significant advantage of the GFATS method, as it can be utilised without the need to adjust its configuration for each individual lens–camera system. While this method formally requires two parameters, *s* and α, the conducted analysis demonstrates that the settings s∈{6,7,8} and α∈{0.05,0.1,0.15} were sufficiently universal across all the tested cases. Within these ranges, the configuration s=6 and α=0.1 consistently provides high-quality results and can be recommended as a standard default. Consequently, the availability of such stable settings eliminates the need for manual adjustment, enabling the GFATS method to operate as a fully automated solution without further user intervention.

## 5. Conclusions

In this paper, the GFATS method for vignetting estimation was introduced. The proposed approach combines the structural reliability of polynomial models with the local adaptability of Gaussian filtering. Through testing across various lens–camera systems, it was demonstrated that GFATS consistently outperforms current state-of-the-art methods, such as DRP, P2D, and SNILP. The primary advantage of this method lies in its ability to fit irregular vignetting profiles accurately without overfitting. This balance is achieved by tuning the σ parameter of the Gaussian filter to an assumed polynomial model M estimated from the central region of the analysed image.

Furthermore, it was found that the GFATS method maintains a high parameter stability. This allows for the definition of a recommended set of parameters (i.e., α and *s*) which, based on experimental results, deliver highly accurate results across different hardware—surpassing the compared model-based methods—without the requirement for manual recalibration. Due to its robustness and fitting flexibility, GFATS serves as a practical, quasi-automated solution for image vignetting correction, particularly for systems characterised by irregular vignetting profiles.

Future research will focus on evaluating the GFATS method across a broader range of imaging systems—such as UAV-mounted sensors, embedded imaging systems, and tilt-shift lenses—to further validate its generalisability and assess the universality of the proposed default parameter set. Since the proposed approach is a non-parametric estimation method, the resulting vignetting estimate $\hat{V}$ is represented as a matrix with the same dimensions as the analysed input image $I_{f}$. Minimising the memory footprint of calibration data is essential for practical applications. Subsequent efforts will therefore investigate techniques to reduce the data volume of the vignetting estimate while maintaining a high correction quality.

## Figures and Tables

**Table 1:** The major parameters of the lens–camera systems used in the experiment.

Parameter	Lens–Camera Set		
	WC_AM_	IC_AM_	DC_AM_
Camera	Logitech C920 (Logitech, Lausanne, Switzerland)	Basler acA1300-30gm (Basler AG, Ahrensburg, Germany)	Canon EOS 650D (Canon Inc., Tokyo, Japan)
Lens		Omron 3Z4S-LE SV-0814H (Omron Corporation, Kyoto, Japan)	Sigma 12–24 mm 1:4.5–5.6 DG HSM (Sigma Corporation, Kawasaki, Japan)
Sensor resolution	1920×1080	1280×960	5184×3456
Sensor type	colour	monochrome	colour
Focal length	unknown	12 mm	12 mm
Aperture		f/1.4	f/4.5

**Table 3:** Comparison of quality measures for vignetting estimation results of the WCam system.

	**Estimation Method**			**Polynomial Order** *s*										
				**2**	**3**	**4**	**5**	**6**	**7**	**8**	**9**	**10**	**11**	**12**
$\mathrm{STD}\left({\hat{I}}_{f}\right)$	DRP			0.3813	0.3761	0.3761	0.3734	0.3698	0.3698	0.3700	0.3702	0.3697	0.3696	0.3693
	P2D			0.3666	0.2695	0.2166	0.2055	0.1888	0.1877	0.1846	0.1837	0.1792	0.3774	0.1921
	SNILP			0.3234	0.2471	0.2037	0.1910	0.1844	0.1821	0.1778	0.1757	0.1743	0.1701	0.1653
	GFATS	α	0.01	0.1508	0.1479	0.1458	0.1439	0.1419	0.1363	0.1363	0.1320	0.1301	0.1279	0.1253
			0.025	0.1540	0.1518	0.1490	0.1463	0.1458	0.1446	0.1439	0.1426	0.1411	0.1385	0.1375
			0.05	0.1577	0.1556	0.1540	0.1518	0.1494	0.1485	0.1479	0.1463	0.1463	0.1452	0.1446
			0.1	0.1635	0.1602	0.1581	0.1569	0.1552	0.1508	0.1494	0.1490	0.1479	0.1474	0.1463
			0.15	0.1622	0.1618	0.1606	0.1593	0.1581	0.1569	0.1552	0.1513	0.1499	0.1499	0.1490
			0.25	0.1643	0.1639	0.1614	0.1610	0.1606	0.1602	0.1589	0.1585	0.1569	0.1544	0.1535
			0.5	0.1652	0.1656	0.1610	0.1598	0.1606	0.1602	0.1593	0.1589	0.1585	0.1577	0.1577
$\mathrm{IQR}\left({\hat{I}}_{f}\right)$	DRP			0.4390	0.4313	0.4311	0.4228	0.4197	0.4200	0.4202	0.4206	0.4195	0.4197	0.4184
	P2D			0.4497	0.3354	0.2801	0.2658	0.2469	0.2448	0.2406	0.2394	0.2323	0.5529	0.2441
	SNILP			0.4279	0.3142	0.2692	0.2515	0.2407	0.2368	0.2309	0.2276	0.2250	0.2194	0.2153
	GFATS	α	0.01	0.1931	0.1898	0.1872	0.1851	0.1827	0.1758	0.1758	0.1704	0.1680	0.1652	0.1619
			0.025	0.1965	0.1941	0.1910	0.1879	0.1872	0.1859	0.1851	0.1836	0.1817	0.1785	0.1772
			0.05	0.2008	0.1985	0.1965	0.1941	0.1915	0.1904	0.1898	0.1879	0.1879	0.1866	0.1859
			0.1	0.2075	0.2038	0.2013	0.1999	0.1980	0.1931	0.1915	0.1910	0.1898	0.1892	0.1879
			0.15	0.2061	0.2056	0.2042	0.2028	0.2013	0.1999	0.1980	0.1936	0.1920	0.1920	0.1910
			0.25	0.2084	0.2080	0.2051	0.2047	0.2042	0.2038	0.2023	0.2018	0.1999	0.1970	0.1961
			0.5	0.2094	0.2099	0.2047	0.2033	0.2042	0.2038	0.2028	0.2023	0.2018	0.2008	0.2008
$R^{2}\left({\hat{I}}_{f}\right)$	DRP			0.9880	0.9883	0.9883	0.9886	0.9887	0.9887	0.9887	0.9887	0.9887	0.9887	0.9887
	P2D			0.9888	0.9940	0.9961	0.9965	0.9970	0.9970	0.9971	0.9972	0.9973	0.9881	0.9969
	SNILP			0.9912	0.9949	0.9965	0.9969	0.9971	0.9972	0.9973	0.9974	0.9975	0.9976	0.9977
	GFATS	α	0.01	0.9981	0.9982	0.9982	0.9983	0.9983	0.9984	0.9984	0.9985	0.9986	0.9986	0.9987
			0.025	0.9980	0.9981	0.9981	0.9982	0.9982	0.9982	0.9983	0.9983	0.9983	0.9984	0.9984
			0.05	0.9979	0.9980	0.9980	0.9981	0.9981	0.9981	0.9982	0.9982	0.9982	0.9982	0.9982
			0.1	0.9977	0.9978	0.9979	0.9979	0.9980	0.9981	0.9981	0.9981	0.9982	0.9982	0.9982
			0.15	0.9978	0.9978	0.9978	0.9978	0.9979	0.9979	0.9980	0.9981	0.9981	0.9981	0.9981
			0.25	0.9977	0.9977	0.9978	0.9978	0.9978	0.9978	0.9979	0.9979	0.9979	0.9980	0.9980
			0.5	0.9977	0.9976	0.9978	0.9978	0.9978	0.9978	0.9978	0.9979	0.9979	0.9979	0.9979

**Note:** The colour scale represents the ranking of the vignetting estimation methods, determined independently for each quality measure and each specified value of *s*. 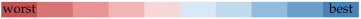

**Table 4:** Comparison of quality measures for vignetting estimation results of the ICam system.

	**Estimation Method**			**Polynomial Order** *s*										
				**2**	**3**	**4**	**5**	**6**	**7**	**8**	**9**	**10**	**11**	**12**
$\mathrm{STD}\left({\hat{I}}_{f}\right)$	DRP			0.9672	0.9670	0.9651	0.9644	0.9635	0.9636	0.9644	0.9630	0.9630	0.9636	0.9638
	P2D			1.0514	1.0250	0.9524	0.9503	0.9253	0.9236	0.9208	0.9172	0.9148	0.9139	0.9147
	SNILP			1.0091	0.9818	0.9384	0.9368	0.9222	0.9200	0.9183	0.9154	0.9143	0.9129	0.9125
	GFATS	α	0.01	0.9123	0.9111	0.9111	0.9111	0.9099	0.9095	0.9085	0.9080	0.9073	0.9073	0.9065
			0.025	0.9126	0.9119	0.9115	0.9115	0.9111	0.9111	0.9111	0.9108	0.9103	0.9099	0.9095
			0.05	0.9155	0.9141	0.9134	0.9126	0.9123	0.9123	0.9119	0.9119	0.9115	0.9115	0.9111
			0.1	0.9199	0.9179	0.9144	0.9137	0.9134	0.9134	0.9130	0.9126	0.9126	0.9126	0.9123
			0.15	0.9233	0.9223	0.9169	0.9152	0.9144	0.9141	0.9137	0.9137	0.9137	0.9134	0.9130
			0.25	0.9270	0.9251	0.9192	0.9189	0.9165	0.9155	0.9148	0.9141	0.9141	0.9141	0.9137
			0.5	0.9380	0.9360	0.9270	0.9270	0.9206	0.9182	0.9176	0.9176	0.9172	0.9172	0.9162
$\mathrm{IQR}\left({\hat{I}}_{f}\right)$	DRP			1.3038	1.3034	1.3012	1.3001	1.2991	1.2992	1.3002	1.2983	1.2984	1.2987	1.2989
	P2D			1.4065	1.3729	1.2792	1.2756	1.2462	1.2454	1.2404	1.2355	1.2329	1.2316	1.2322
	SNILP			1.3582	1.3222	1.2628	1.2598	1.2411	1.2390	1.2364	1.2337	1.2318	1.2296	1.2297
	GFATS	α	0.01	1.2289	1.2276	1.2276	1.2276	1.2264	1.2259	1.2248	1.2240	1.2231	1.2231	1.2221
			0.025	1.2292	1.2286	1.2282	1.2282	1.2276	1.2276	1.2276	1.2272	1.2268	1.2264	1.2259
			0.05	1.2330	1.2311	1.2301	1.2292	1.2289	1.2289	1.2286	1.2286	1.2282	1.2282	1.2276
			0.1	1.2376	1.2357	1.2317	1.2306	1.2301	1.2301	1.2297	1.2292	1.2292	1.2292	1.2289
			0.15	1.2414	1.2403	1.2346	1.2326	1.2317	1.2311	1.2306	1.2306	1.2306	1.2301	1.2297
			0.25	1.2455	1.2432	1.2372	1.2367	1.2342	1.2330	1.2322	1.2311	1.2311	1.2311	1.2306
			0.5	1.2569	1.2545	1.2455	1.2455	1.2383	1.2361	1.2353	1.2353	1.2350	1.2350	1.2339
$R^{2}\left({\hat{I}}_{f}\right)$	DRP			0.9429	0.9429	0.9430	0.9430	0.9430	0.9430	0.9430	0.9430	0.9430	0.9430	0.9430
	P2D			0.9304	0.9338	0.9435	0.9438	0.9470	0.9472	0.9476	0.9480	0.9484	0.9485	0.9484
	SNILP			0.9361	0.9394	0.9454	0.9455	0.9475	0.9478	0.9480	0.9484	0.9485	0.9486	0.9487
	GFATS	α	0.01	0.9488	0.9490	0.9490	0.9490	0.9491	0.9492	0.9493	0.9494	0.9494	0.9494	0.9495
			0.025	0.9488	0.9489	0.9489	0.9489	0.9490	0.9490	0.9490	0.9490	0.9491	0.9491	0.9492
			0.05	0.9485	0.9486	0.9487	0.9488	0.9488	0.9488	0.9489	0.9489	0.9489	0.9489	0.9490
			0.1	0.9480	0.9482	0.9486	0.9487	0.9487	0.9487	0.9488	0.9488	0.9488	0.9488	0.9488
			0.15	0.9475	0.9477	0.9483	0.9485	0.9486	0.9486	0.9487	0.9487	0.9487	0.9487	0.9488
			0.25	0.9471	0.9473	0.9480	0.9481	0.9484	0.9485	0.9486	0.9486	0.9486	0.9486	0.9487
			0.5	0.9456	0.9459	0.9471	0.9471	0.9479	0.9482	0.9482	0.9482	0.9483	0.9483	0.9484

**Note:** The colour scale represents the ranking of the vignetting estimation methods, determined independently for each quality measure and each specified value of *s*. 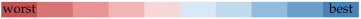

**Table 5:** Comparison of quality measures for vignetting estimation results of the DCam system.

	**Estimation Method**			**Polynomial Order** *s*										
				**2**	**3**	**4**	**5**	**6**	**7**	**8**	**9**	**10**	**11**	**12**
$\mathrm{STD}\left({\hat{I}}_{f}\right)$	DRP			0.9632	0.9505	0.9505	0.9418	0.9407	0.9413	0.9404	0.9398	0.9396	0.9402	0.9392
	P2D			0.9623	0.9265	0.8231	0.8114	0.7742	0.7689	0.7315	0.7262	0.7654	0.7136	0.7314
	SNILP			0.9042	0.8834	0.7925	0.7887	0.7354	0.7313	0.7166	0.7102	0.7030	0.7006	0.6968
	GFATS	α	0.01	0.6838	0.6834	0.6822	0.6797	0.6777	0.6766	0.6740	0.6731	0.6714	0.6702	0.6695
			0.025	0.6856	0.6825	0.6822	0.6816	0.6804	0.6801	0.6797	0.6788	0.6784	0.6773	0.6758
			0.05	0.6904	0.6875	0.6853	0.6840	0.6828	0.6828	0.6825	0.6816	0.6810	0.6807	0.6804
			0.1	0.6944	0.6940	0.6918	0.6884	0.6871	0.6862	0.6853	0.6853	0.6847	0.6847	0.6844
			0.15	0.6972	0.6956	0.6937	0.6925	0.6911	0.6891	0.6878	0.6871	0.6865	0.6862	0.6859
			0.25	0.6980	0.6980	0.6940	0.6933	0.6925	0.6918	0.6911	0.6897	0.6888	0.6881	0.6871
			0.5	0.7365	0.7351	0.7255	0.7175	0.7024	0.6968	0.6908	0.6881	0.6853	0.6853	0.6850
$\mathrm{IQR}\left({\hat{I}}_{f}\right)$	DRP			1.2059	1.1660	1.1684	1.1549	1.1524	1.1536	1.1539	1.1530	1.1539	1.1546	1.1535
	P2D			1.2140	1.1236	1.0675	1.0536	0.9710	0.9652	0.9116	0.8994	0.9403	0.8820	0.9015
	SNILP			1.1143	1.0816	1.0122	1.0091	0.9247	0.9159	0.8877	0.8775	0.8678	0.8649	0.8594
	GFATS	α	0.01	0.8413	0.8410	0.8398	0.8371	0.8348	0.8336	0.8309	0.8298	0.8280	0.8267	0.8260
			0.025	0.8432	0.8401	0.8398	0.8391	0.8378	0.8374	0.8371	0.8360	0.8356	0.8344	0.8328
			0.05	0.8480	0.8451	0.8429	0.8416	0.8404	0.8404	0.8401	0.8391	0.8385	0.8382	0.8378
			0.1	0.8518	0.8514	0.8494	0.8460	0.8448	0.8438	0.8429	0.8429	0.8423	0.8423	0.8420
			0.15	0.8542	0.8528	0.8511	0.8500	0.8487	0.8467	0.8454	0.8448	0.8441	0.8438	0.8435
			0.25	0.8550	0.8550	0.8514	0.8507	0.8500	0.8494	0.8487	0.8473	0.8464	0.8458	0.8448
			0.5	0.8837	0.8828	0.8763	0.8705	0.8587	0.8539	0.8484	0.8458	0.8429	0.8429	0.8426
$R^{2}\left({\hat{I}}_{f}\right)$	DRP			0.9960	0.9962	0.9962	0.9962	0.9962	0.9962	0.9962	0.9962	0.9962	0.9962	0.9962
	P2D			0.9960	0.9963	0.9970	0.9971	0.9974	0.9975	0.9977	0.9977	0.9975	0.9978	0.9977
	SNILP			0.9965	0.9967	0.9973	0.9973	0.9977	0.9977	0.9978	0.9978	0.9979	0.9979	0.9979
	GFATS	α	0.01	0.9980	0.9980	0.9980	0.9980	0.9980	0.9980	0.9980	0.9981	0.9981	0.9981	0.9981
			0.025	0.9980	0.9980	0.9980	0.9980	0.9980	0.9980	0.9980	0.9980	0.9980	0.9980	0.9980
			0.05	0.9980	0.9980	0.9980	0.9980	0.9980	0.9980	0.9980	0.9980	0.9980	0.9980	0.9980
			0.1	0.9979	0.9979	0.9979	0.9980	0.9980	0.9980	0.9980	0.9980	0.9980	0.9980	0.9980
			0.15	0.9979	0.9979	0.9979	0.9979	0.9979	0.9980	0.9980	0.9980	0.9980	0.9980	0.9980
			0.25	0.9979	0.9979	0.9979	0.9979	0.9979	0.9979	0.9979	0.9980	0.9980	0.9980	0.9980
			0.5	0.9977	0.9977	0.9977	0.9978	0.9979	0.9979	0.9979	0.9980	0.9980	0.9980	0.9980

**Note:** The colour scale represents the ranking of the vignetting estimation methods, determined independently for each quality measure and each specified value of *s*. 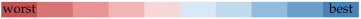

**Table 6:** The values of $\sigma^{★}$ obtained using the GFATS method for WCam.

α	**Polynomial Order** *s*										
	**2**	**3**	**4**	**5**	**6**	**7**	**8**	**9**	**10**	**11**	**12**
0.01	20	17	15	13.5	12	9	9	7.5	7	6.5	6
0.025	23.5	21	18	15.5	15	14	13.5	12.5	11.5	10	9.5
0.05	28	25.5	23.5	21	18.5	17.5	17	15.5	15.5	14.5	14
0.1	35	31	28.5	27	25	20	18.5	18	17	16.5	15.5
0.15	33.5	33	31.5	30	28.5	27	25	20.5	19	19	18
0.25	36	35.5	32.5	32	31.5	31	29.5	29	27	24	23
0.5	37	37.5	32	30.5	31.5	31	30	29.5	29	28	28

**Table 7:** The values of $\sigma^{★}$ obtained using the GFATS method for ICam.

α	**Polynomial Order** *s*										
	**2**	**3**	**4**	**5**	**6**	**7**	**8**	**9**	**10**	**11**	**12**
0.01	11	9.5	9.5	9.5	8	7.5	6.5	6	5.5	5.5	5
0.025	11.5	10.5	10	10	9.5	9.5	9.5	9	8.5	8	7.5
0.05	15.5	13.5	12.5	11.5	11	11	10.5	10.5	10	10	9.5
0.1	22	19	14	13	12.5	12.5	12	11.5	11.5	11.5	11
0.15	27	25.5	17.5	15	14	13.5	13	13	13	12.5	12
0.25	32	29.5	21	20.5	17	15.5	14.5	13.5	13.5	13.5	13
0.5	44	42	32	32	23	19.5	18.5	18.5	18	18	16.5

**Table 8:** The values of $\sigma^{★}$ obtained using the GFATS method for DCam.

α	**Polynomial Order** *s*										
	**2**	**3**	**4**	**5**	**6**	**7**	**8**	**9**	**10**	**11**	**12**
0.01	37.5	37	35	31	28	26.5	23.5	22.5	21	20	19.5
0.025	40.5	35.5	35	34	32	31.5	31	29.5	29	27.5	25.5
0.05	48	43.5	40	38	36	36	35.5	34	33	32.5	32
0.1	53.5	53	50	45	43	41.5	40	40	39	39	38.5
0.15	57	55	52.5	51	49	46	44	43	42	41.5	41
0.25	58	58	53	52	51	50	49	47	45.5	44.5	43
0.5	92	91	84	77.5	63	56.5	48.5	44.5	40	40	39.5

**Table 9:** Comparison of the spread of STD values.

**System**	**Estimation Method**	min(STD)	max(STD)	d(STD)	D(STD) [%]	$\frac{d(\mathrm{STD})}{d_{\mathrm{GFATS}}\left(\mathrm{STD}\right)}$	$\frac{D(\mathrm{STD})}{D_{\mathrm{GFATS}}\left(\mathrm{STD}\right)}$
WCam	DRP	0.3693	0.3813	0.0120	3.25	0.30	0.10
	P2D	0.1792	0.3774	0.1983	110.66	4.92	3.44
	SNILP	0.1653	0.3234	0.1581	95.64	3.92	2.97
	GFATS	0.1253	0.1656	0.0403	32.15	1.00	1.00
ICam	DRP	0.9630	0.9672	0.0042	0.44	0.13	0.13
	P2D	0.9139	1.0514	0.1374	15.04	4.37	4.33
	SNILP	0.9125	1.0091	0.0966	10.59	3.07	3.05
	GFATS	0.9065	0.9380	0.0315	3.47	1.00	1.00
DCam	DRP	0.9392	0.9632	0.0241	2.56	0.36	0.26
	P2D	0.7136	0.9623	0.2486	34.84	3.71	3.48
	SNILP	0.6968	0.9042	0.2074	29.77	3.10	2.97
	GFATS	0.6695	0.7365	0.0670	10.01	1.00	1.00

**Table 10:** Comparison of the spread of IQR values.

**System**	**Estimation Method**	min(IQR)	max(IQR)	d(IQR)	D(IQR) [%]	$\frac{d(\mathrm{IQR})}{d_{\mathrm{GFATS}}\left(\mathrm{IQR}\right)}$	$\frac{D(\mathrm{IQR})}{D_{\mathrm{GFATS}}\left(\mathrm{IQR}\right)}$
WCam	DRP	0.4184	0.4390	0.0205	4.91	0.43	0.17
	P2D	0.2323	0.5529	0.3206	137.98	6.67	4.65
	SNILP	0.2153	0.4279	0.2126	98.76	4.43	3.33
	GFATS	0.1619	0.2099	0.0480	29.67	1.00	1.00
ICam	DRP	1.2983	1.3038	0.0055	0.42	0.16	0.15
	P2D	1.2316	1.4065	0.1750	14.21	5.03	4.99
	SNILP	1.2296	1.3582	0.1287	10.46	3.70	3.67
	GFATS	1.2221	1.2569	0.0348	2.85	1.00	1.00
DCam	DRP	1.1524	1.2059	0.0535	4.64	0.93	0.66
	P2D	0.8820	1.2140	0.3320	37.64	5.75	5.39
	SNILP	0.8594	1.1143	0.2549	29.66	4.42	4.24
	GFATS	0.8260	0.8837	0.0577	6.99	1.00	1.00

**Figure A1:**
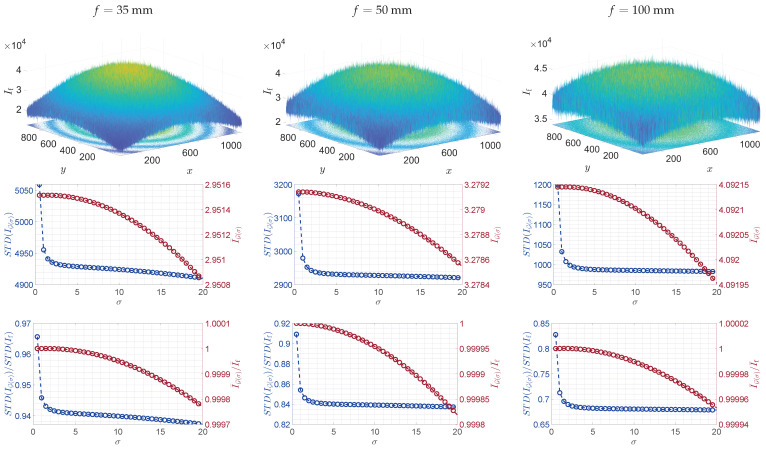
Comparison of the influence of the σ parameter on the selection criteria proposed in [44] for simulated $I_{f}$ images. **Top row**: simulated $I_{f}$ images for three different focal lengths *f*. **Middle row**: plots of the standard deviation $\mathrm{STD}(I_{G(\sigma )})$ and the mean value ${\bar{I}}_{G(\sigma )}$ for varying σ values. **Bottom row**: plots of $\mathrm{STD}\left(I_{G(\sigma )}\right)/\mathrm{STD}\left(I_{f}\right)$ and ${\bar{I}}_{G(\sigma )}/{\bar{I}}_{f}$ as a function of the σ parameter.

## Data Availability

Data from this study and the MATLAB code for vignetting estimation are available from the authors upon reasonable request.

## Abbreviations

The following abbreviations are used in this manuscript:

CRF	**C**amera **r**esponse **f**unction
DFO	**D**erivative-**f**ree **o**ptimization technique
DRP	**D**eformable **r**adial **p**olynomial vignetting model
FFT	**F**ast **F**ourier **t**ransform
GAN	**G**enerative **a**dversarial **n**etwork
GFATS	**G**aussian **f**ilter with **a**uto-**t**uned **s**igma vignetting estimation method
GFWH	**G**aussian **f**ilter **w**ith **h**armony method
HDR	**H**igh-**d**ynamic-**r**ange imaging
IQR	**I**nter**q**uartile **r**ange
MSE	**M**ean **s**quared **e**rror
P2D	**P**olynomial **2D** vignetting model
PMMA	**P**oly(**m**ethyl **m**eth**a**crylate)
SNILP	**S**mooth **n**on-**i**terative **l**ocal **p**olynomial vignetting model
STD	**St**andard **d**eviation

## Appendix A. Analysis of the Gaussian Filter with Harmony Method Proposed by Cao et al.

### Appendix A.1. Method Overview

As mentioned in Section 1, the σ value selection procedure employed in the Gaussian filter with harmony (GFWH) method proposed by Cao et al. in [44] is based on a comparison of the standard deviation $\mathrm{STD}\left(I_{G(\sigma )}\right)$ and the mean value ${\bar{I}}_{G(\sigma )}$ of the filtered image $I_{G(\sigma )}$ with the corresponding $\mathrm{STD}(I_{f})$ and ${\bar{I}}_{f}$ values of the input image $I_{f}$. The image $I_{G(\sigma )}$ is obtained by applying the filter G(σ) to $I_{f}$. According to the authors (Equation (6) in [44]), the optimal σ value, denoted as $\sigma^{★}$, must satisfy the following condition:

(A1) $$\sigma^{★}=\arg\min_{\sigma }\left(\frac{\mathrm{STD}\left(I_{G(\sigma )}\right)}{\mathrm{STD}\left(I_{f}\right)}>0.99\;\wedge \;\frac{{\bar{I}}_{G(\sigma )}}{{\bar{I}}_{f}}>0.99\right).$$

An analysis of Figures 7, 11, and 15 in [44], which illustrate the dependence of the ${\bar{I}}_{G(\sigma )}$ and $\mathrm{STD}\left(I_{G(\sigma )}\right)$ measures on σ, revealed that these curves increase monotonically. Furthermore, these plots exhibit two distinct regions: an initial phase of rapid growth followed by a second region characterised by asymptotic behaviour. An examination of the GFWH method’s flowchart (Figure 10 in [44]), the condition in (A1), and the shape of the ${\bar{I}}_{G(\sigma )}$ and $\mathrm{STD}\left(I_{G(\sigma )}\right)$ curves revealed that this behaviour is a necessary condition for the method’s successful application.

### Appendix A.2. Experimental Validation

To evaluate the applicability of the criterion defined in (A1), the effect of Gaussian filtering on simulated vignetting images was investigated. To ensure consistency with the data used in [44], simulated images $I_{f}$ were generated at a resolution of 1280×960 pixels, with the optical centre (corresponding to the minimum vignetting intensity) located at coordinates $C=\left(i_{C},j_{C}\right)=\left(664,545\right)$. Vignetting was simulated using a non-radial version of the natural vignetting model (cos^4^ law) as described in [32]. In this model, the degree of non-radiality is represented by the parameter η (see Section 3.2.3 or [34]); for this simulation, η=1.1 was adopted.

Vignetting simulations were performed for three distinct lens focal lengths: 35 mm, 50 mm, and 100 mm. To obtain the $I_{f}$ images, the simulated vignetting V∈(0,1) was contaminated with mixed noise and rescaled to a range of 0–47,000, consistent with the pixel values reported by Cao et al. This process is defined as follows:

(A2) $$\begin{array}{cc} V_{\mathrm{noise}}\left(i,j\right) & =N\left(1,\sigma_{*}\right)\;\cdot \;V\left(i,j\right)+N\left(0,\sigma_{+}\right), \end{array}$$

(A3) $$\begin{array}{cc} I_{f} & =L_{max}\;\cdot \;\frac{V_{\mathrm{noise}}}{\max(V_{\mathrm{noise}})}, \end{array}$$

where $N(\mu_{N},\sigma_{N})$ denotes a random value from a normal distribution with mean $\mu_{N}$ and standard deviation $\sigma_{N}$. In the simulation, $L_{max}=47,000$, while $\sigma_{+}=0.05$ and $\sigma_{*}=0.025$ were used for the additive and multiplicative noise components, respectively.

The simulation results are presented in the top row of Figure A1, while the middle row illustrates the standard deviation $\mathrm{STD}\left(I_{G(\sigma )}\right)$ and the mean ${\bar{I}}_{G(\sigma )}$ of the filtering results $I_{G(\sigma )}$ for σ∈{0.5,1,…,20}.

A comparison of these results with the plots in Figures 7, 11, and 15 in [44] revealed a significant discrepancy. Specifically, both the $\mathrm{STD}\left(I_{G(\sigma )}\right)$ and ${\bar{I}}_{G(\sigma )}$ measures decrease monotonically as the value of σ increases. This fundamental change in the relationship between these measures and the parameter σ renders the application of criterion (A1) ineffective. Consequently, in the cases investigated here, applying this criterion simply returns the smallest considered σ value, leading to evident overfitting of the estimate to the noisy data.

Furthermore, comparing the $\mathrm{STD}\left(I_{G(\sigma )}\right)$ and ${\bar{I}}_{G(\sigma )}$ with the parameters of the input image $I_{f}$ (bottom row of Figure A1) revealed that the first part of the condition in (A1), i.e., $\mathrm{STD}\left(I_{G(\sigma )}\right)/\mathrm{STD}\left(I_{f}\right)>0.99$, is not satisfied for any of the considered σ values. Conversely, the second part of the condition, i.e., ${\bar{I}}_{G(\sigma )}/{\bar{I}}_{f}>0.99$, is satisfied for every σ value under consideration. A clear contradiction therefore exists between both parts of the condition, rendering the method impossible to apply to the tested cases.

### Appendix A.3. Remarks on the Applicability of the GFWH Method

The simulation results demonstrate that the GFWH method for σ selection proposed by Cao et al. is unsuitable for the data investigated in this study, as the statistical measures do not satisfy the criterion in Equation (A1). Consequently, this method was not utilised in our primary research. The exact causes of the discrepancy between our findings and those of Cao et al. have yet to be determined.
